# Altered local RAS in the liver increased the risk of NAFLD in male mouse offspring produced by in vitro fertilization

**DOI:** 10.1186/s12884-023-05681-8

**Published:** 2023-05-12

**Authors:** Le Bo, Lun Wei, Linling Shi, Chao Luo, Shasha Gao, Anwen Zhou, Caiping Mao

**Affiliations:** 1 Reproductive Medicine Center, First Affiliated Hospital of Soochow University, No.899 Pinghai Road, Suzhou, Jiangsu 215000 China; 2 Department of Gynaecology and Obstetrics, First Affiliated Hospital of Soochow University, Suzhou, Jiangsu 215000 China

**Keywords:** In vitro fertilization, Renin-angiotensin system, Nonalcoholic fatty liver disease, High-fat diet, Metabolism.

## Abstract

**Background:**

Assisted reproductive technology (ART) is associated with an increased risk of adverse metabolic health in offspring, and these findings have been demonstrated in animal models without parental infertility issues. However, it is unclear what changes lead to abnormal metabolism. The activation of the renin-angiotensin system (RAS) has been related to various aspects of metabolic syndrome. Thus, we focused on the local RAS of the liver, which is the central organ for glucose and lipid metabolism in offspring conceived by in vitro fertilization (IVF), and studied the role of local liver RAS in metabolic diseases.

**Methods:**

Male C57BL/6 mouse offspring obtained by natural pregnancy and IVF were fed a standard chow diet or a high-fat diet (HFD) from 4 weeks of age through 16 weeks of age. We assessed glucose and lipid metabolism, hepatic histopathology, and the gene and protein expression of key RAS components. In addition, the blocker losartan was used from 4 weeks of age through 16 weeks of age to investigate the regulatory mechanisms of abnormal local RAS on metabolic activity in the IVF offspring liver.

**Results:**

The growth trajectories of IVF offspring body and liver weights were different from those of naturally pregnant offspring. Impaired glucose tolerance (IGT) and insulin resistance (IR) occurred in IVF-conceived male offspring. After continuous HFD feeding, male offspring in the IVF group underwent earlier and more severe IR. Furthermore, there was a trend of lipid accumulation in the livers of chow-fed IVF offspring. Hepatic steatosis was also more serious in the IVF offspring after HFD treatment. Type 1 receptor (AT1R), which is the primary receptor mediating the action of angiotensin (Ang) II, has been confirmed to be upregulated in IVF offspring livers. Losartan reduced or even eliminated most of the significant differences between the IVF and NC groups after HFD consumption.

**Conclusions:**

The upregulation of AT1R expression in the liver increased the activity of the local RAS, resulting in abnormal glucose and lipid metabolism and lipid accumulation in the liver, significantly increasing the risk of nonalcoholic fatty liver disease (NAFLD) in IVF offspring.

**Supplementary Information:**

The online version contains supplementary material available at 10.1186/s12884-023-05681-8.

## Introduction

More than 8 million children have been conceived by the development of ART worldwide [1]. Despite wide acceptance and implementation of ART as a treatment for many common forms of infertility, concerns have been expressed about the safety of ART’s effects on the child’s development. More recently, ART has been associated with long-term health concerns in adult offspring, including increased fat deposition, growth velocity, cardiovascular dysfunction and elevated blood pressure [2–4].

Developmental origins of health and disease (DOHaD) is the hypothesis relating perinatal origins to adult diseases. The early life environment may have long-lasting consequences on health, according to the DOHaD [5, 6]. IVF procedures and manipulations occur during the process of preimplantation development, and DNA methylation and histone modifications undergo substantial changes, which regulate specific and heritable patterns of gene expression. The long-term healthy evidence is limited, since most IVF offspring are less than 40 years old. Nevertheless, epidemiological evidence of abnormal glucose and lipid metabolism has been identified in human cohort studies of children and young adults conceived with IVF [7, 8]. A deprived developmental environment sets up an individual’s metabolism for nutritional scarcity, according to the mismatch hypothesis within the DOHaD framework.

To distinguish the consequences mediated through parental infertility and ART practice, mouse models have inestimable value in evaluating the effects of ART-related techniques on the long-term health of offspring, which could eliminate the effects of genetic and environmental factors. These studies in mice show that IVF leads to different growth trajectories, IGT, hyperinsulinaemia and abnormal lipid metabolism [9, 10].

NAFLD can be classified as simple steatosis (NAFL) and nonalcoholic steatohepatitis (NASH) and has tended to become epidemic in children worldwide in the last decade [11]. Although the pathogenesis of NAFLD is still unclear, the ”2-hit hypothesis” is widely accepted [12]. The theory suggests that suboptimal conditions and adaptations in early development (first-hit) may lay the foundation for long-term susceptibility to subsequent adverse events (second-hit), which may occur in the foetus or later life, leading to more extreme outcomes or phenotypes [13]. The explanation of NAFLD in the second strike theory is also multifactorial, including epigenetic, metabolic and environmental factors. The RAS has been identified in the liver and is now recognized as an important modulator of body metabolic processes. Lipid accumulation and altered RAS function are thought to play a synergistic role in the process of NAFLD [14].

Based on this model, the present study was devised to assess the combined effects of fertility mode (first-hit) and dietary behaviour (second-hit) on metabolic health, growth and development in offspring liver. More importantly, the “second hit” can enlarge the potential defects and help us understand the risk of disease. Our evidence shows that IVF changed the growth trajectory after birth and impaired glucose and lipid metabolism in offspring. Moreover, after continuous HFD feeding, the risk of NAFLD in IVF offspring increased significantly.

## Materials and methods

### Animals

All experimental procedures with mice were approved by the Soochow University’s Ethical Committee in Animal Research. C57/BL6 mice (donors of gametes) and ICR females (pseudopregnancy recipients) were bred in-house (Laboratory Animal Center of Soochow University) under a constant 12-h light/12-h dark cycle at 22–24◦C with free access to food and water.

### Generation of IVF offspring

Virgin female C57/BL6 mice (7–8 weeks old) were superovulated by intraperitoneal injection of 5 IU pregnant mare serum gonadotropin (PMSG, Ningbo No.2 Hormone Factory, CN), and then 5 IU human chorionic gonadotropin (HCG, Ningbo No.2 Hormone Factory, CN) after 48 h. 14–15 h after HCG administration, oocytes were collected from the ampullae and fertilized in vitro with sperms from epididymal tail of C57/BL6 male mice (8–9 weeks old). For IVF, Sperms were treated in a pre-balanced (5% CO_2_ 5%O_2_ at 37◦C) medium TYH (Nanjing AIBI bio-Technology Co.Ltd, CN). Gametes were co-incubated in HTF (EasyCheck, Nanjing AIBI bio-Technology Co.Ltd, CN) in a 5% CO_2_ and 5%O_2_ incubator at 37◦C (Labotect, GER). After 6 h of culture, zygotes were transferred to medium KSOM(Nanjing AIBI bio-Technology Co.Ltd, CN) and transplanted as 2-cell embryos. 2.5 days prior to embryo transfer, ICR females (7–8 weeks old) were co-housed with vasectomized ICR males in a 1: 1 ratio. The next morning, the presence of vaginal plugs was considered a sign of success in pseudopregnant females. Fresh 2-cell embryos were transferred in equal numbers into the both oviducts of pseudopregnancy Recipients. The mice were anesthetized by tribromoethanol (Nanjing AIBI bio-Technology Co.Ltd, CN). All recipients were fed with the chow diet.

### Animal treatment groups

**Fig. 1:**
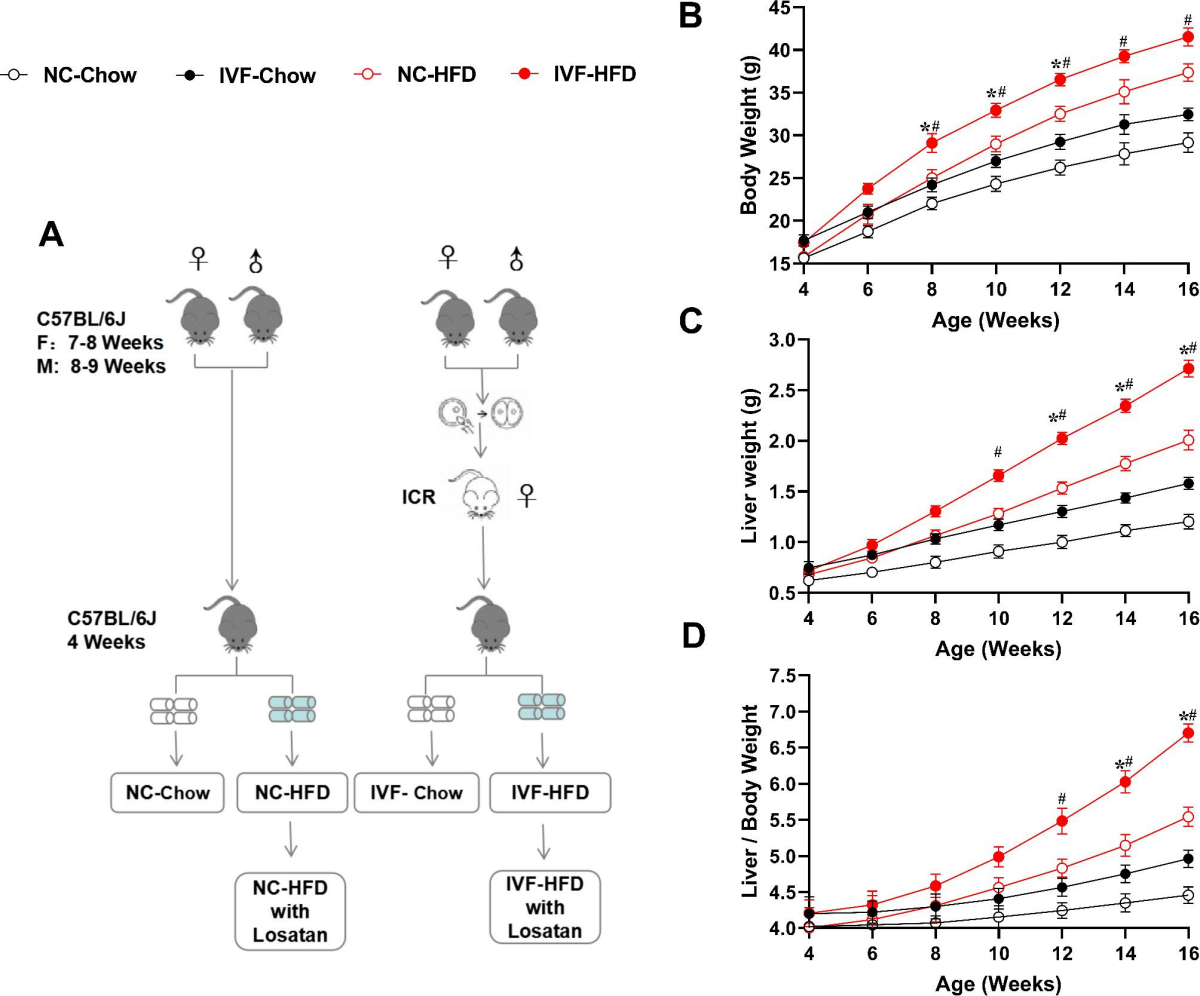
Experimental design and growth curves of male offspring. **A**: Experimental design showing the six treatment groups used. **B**: Body weight in male offspring. **C**: Liver weight in male offspring. **D**: Liver/Body index in male offspring. *Indicates a significant difference(*P* < 0.05) between NC-Chow and IVF-Chow; #denotes NC-HFD vs. IVF-HFD (*P* < 0.05). Mean (± SEM) body weight from 4 to 16 weeks (from 8 to 10 litters). n_NC−Chow_=12, n_IVF−Chow_=12,n_NC−HFD_=10, n_IVF−HFD_=10.

The overall experimental design and grouping situation of this study is shown in the flow chart of Fig. 1A. The control group was the offspring of naturally pregnant mice (NC). All offspring from the five treatment groups were weaned at 4 weeks. After weaning, all the offspring were randomly divided into the chow diet group, the HFD group and the HFD with Losartan intervention group(the intervention group was intragastrically administered with Losartan 5 mg/kg once a day from 4 weeks of age). HFD exposure (nutrient composition 60% fat, 20% protein, and 20% carbohydrate) served as the “second hit” to enlarge the potential defects in disease state. Therefore, six groups were set up after weaning, including NC-Chow group, IVF-Chow group, NC-HFD group, IVF-HFD group, NC-HFD with losartan group and IVF-HFD with losartan group.

### Intraperitoneal glucose tolerance test

Glucose concentration in offspring serum was measured using the glucometer (Accu-Chek Performa, Roche Diagnostics GmbH). And glucose tolerance tests (GTT) were conducted at postnatal 4 weeks in unrestrained conscious mice after 12 h overnight fast, with access to water. Serum glucose levels were measured at 0, 15, 30, 60, 120 min after intraperitoneal injection of glucose solution (2 g/kg) into the mice. FBG and FINS were measured at the same time in the morning, following a 12 h overnight fast. HOMA-IR = FBG×FINS/22.5.

### Liver morphological analysis

Fresh mouse liver tissue was taken as soon as possible, immediately after submersion with OCT and quickly frozen with liquid nitrogen. After that, it is fixed and sliced, and stained with Oil Red O for histopathology. The hepatic histological changes were observed by light microscopy(Olympus, JPN). After HE staining, intracellular lipid droplets are specifically stained red, and the size and distribution of the lipid droplets directly represent the degree of steatosis.

Mouse livers were collected, washed, fixed, and stained with HE for histopathology. The hepatic histological examination was performed using light microscopy (Olympus, JPN). The steatosis in hepatocytes was observed. After HE staining, it showed intracellular vacuoles, and the degree of steatosis was indirectly reflected by the size and distribution of the vacuoles.

### Serum hormone and biochemical test

Blood samples of mice were taken from postcava and centrifuged to get the serum. The ELISA kits (Jiangsu Meimian industrial Co.,Ltd., CN) were used to survey the levels of angiotensinogen(AGT), angiotensin converting enzyme 1(ACE1), angiotensin II(Ang II) and insulin concentration according to the instructions of manufacturers. The biochemical test were used to survey the levels of total cholesterol (TC), triglyceride (TG), low density lipoprotein (LDL), alkaline phosphatase (ALP), aspartate aminotransferase (AST), alanine aminotransferase (ALT).

### Real-time PCR

Total RNA was isolated from offspring livers, and was purified using a protocol adapted from published methods [15]. Messenger RNA (mRNA) abundance of AGT, ACE1, AT1R, AT2R in the liver was determined using quantitative Real-time PCR (Thermo Fisher Scientific, USA). The RNeasy Mini Kits (Qiagen, Germany) were used to extract RNA from mouse livers. RNA was only used if the ratio determined with a spectrophotometer was between 1.8 and 2.0, denoting minimum contamination from cellular proteins. To generate cDNA, 1 µg of RNA with oligo dT and a reverse transcription kit (Transgen Biotech, CN) was used. The primers are presented in Table 1, and FastStart Universal SYBR Green Master (Thermo Fisher Scientific, USA) were used to carry quantitative PCR with the StepOnePlus™ Real-Time PCR System (Thermo Fisher Scientific, USA). β-actin was used as an internal control. All experiments were repeated three times for reliability. The relative gene expression (RGE) was normalized to β-actin to obtain the relative threshold cycle and calculated as RGE = 2 − ΔΔCt.

**Table 1:** Sequences of the primers used in QRT-PCR

Target gene	Primer	Nucleotide sequence
AGT	F	5’-CAGGTCGCAATGATCGCCA-3’
	R	5’-GTGTCCATCTAGTCGGGAGGT-3’
ACE1	F	5’-TGCCAAGCTCAATGGCTACA-3’
	R	5’-GGTCTTGCTCCAGGTTGTCA-3’
AT1R	F R	5’-TGCCATGCCCATAACCATCTG-3’ 5’-CGTGCTCATTTTCGTAGACAGG-3’
AT2R	F R	5’-ACTTTATGAATGGCATCGAGCTT-3’ 5’-GGGGATAAGAACCTAAACAGCAC-3’
β-actin	F R	5’-GTCGTACCACAGGCATTGTGATGG-3’ 5’-GCAATGCCTGGGTACATGGTGG-3’

### Western blot

Mouse livers were lysed by radioimmunoprecipitation assay (RIPA) buffer. Protein abundance of AGT, ACE1, AT1R, AT2R in the liver was measured with western blot analysis normalized to β-actin as described previously [15]. The primary antibodies were diluted (AGT, 1:2000; ACE1,1:1000; AT1R, 1:800; AT2R, 1:1500; β-actin, 1:20000; Affinity, USA) and used, then goat anti-rabbit secondary antibody (1:4000) was used. Blots were developed using enhanced chemiluminescence detection reagents (Absin, CN), and results were analyzed by Image J software.

### Statistical analysis

All data were shown as the mean ± SEM. GraphPad Prism 9.0 was used for statistical analysis of results from the two groups through *t-test* or two-way ANOVA, whereas time courses were analyzed by repeated-measures ANOVA with Bonferroni post hoc analysis, and significance was taken as *P* < 0.05. It was considered to be a trend, if the *P* value is between 0.1 and 0.05. As mice on HFD have different glucose and lipid metabolism patterns, the components exposed to different diets are not compared, only the experimental group and the control group with the same dietary components are compared.

## Results

### Distinct body and liver weight trajectories were shown in IVF offspring

To study the effect of IVF on postnatal development, we generated six treatment groups, as shown in Fig. 1A. The mean litter size of pregnant mice in the NC group (n = 20) was 8.20 ± 0.48, while that in the IVF group (n = 20) was 7.20 ± 0.43. There was no difference in litter size between the two groups (*P* = 0.1226). The sex ratio (male/female) was 1.0 (83:81) in the control group and 1.2 (79:65) in the IVF group.

After weaning, offspring body weights in the IVF-chow group were higher than those in the NC-chow group from 8 weeks through 12 weeks. However, the HFD group maintained a significant difference until the age of 16 weeks (Fig. 1B). Significant differences in the liver weight (Fig. 1C)and the liver index (Fig. 1D) between IVF group and NC group occurred earlier in the treatment of HFD. These results suggest that the IVF-ET process deviated the offspring from the normal developmental trajectory, and HFD made the difference more significant.

### IVF offspring develop impaired glucose and lipid metabolism

**Fig. 2:**
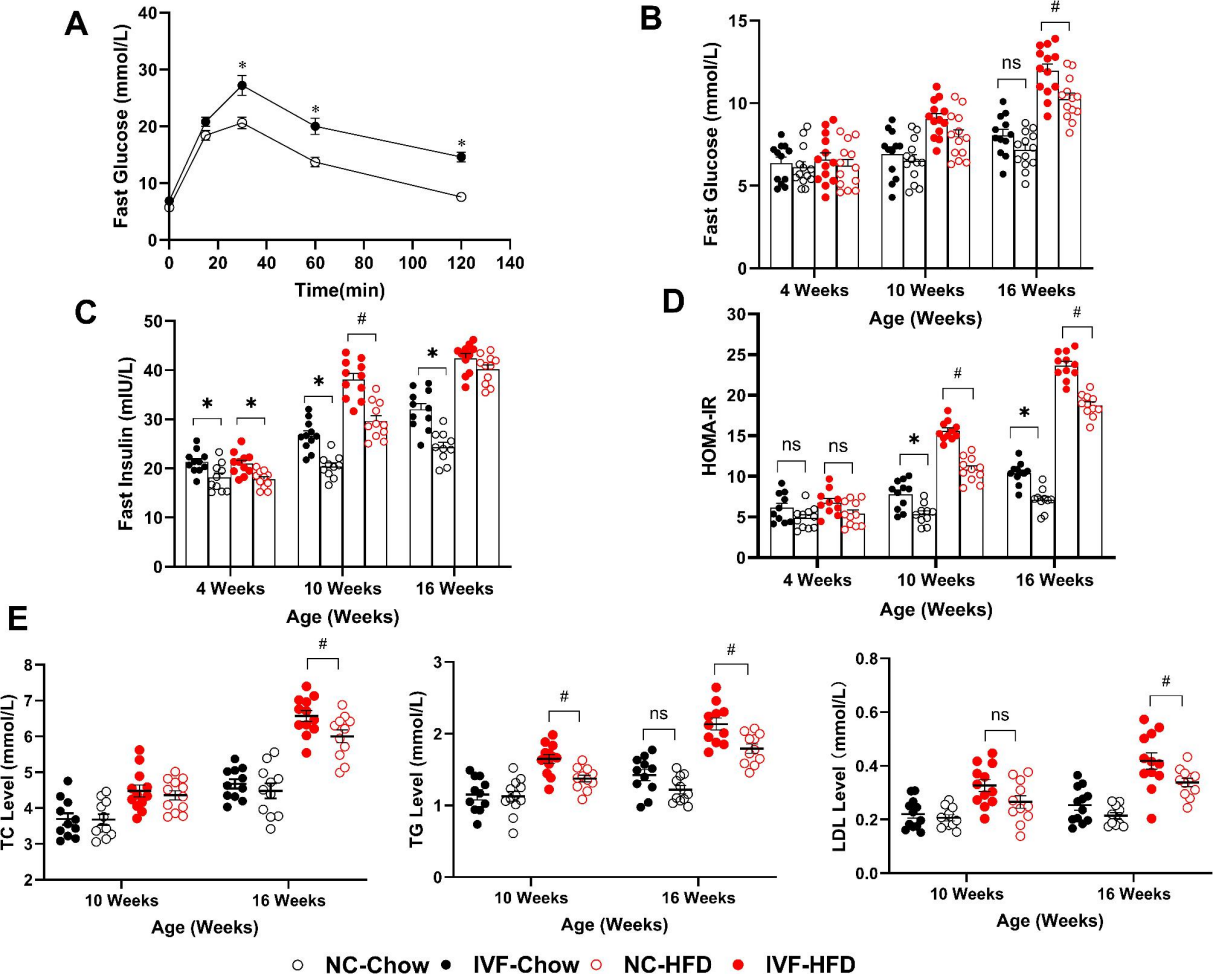
Effect of IVF-ET on glucose and lipid metabolism of male offspring. **A**: Glucose tolerance test in 4-week-old chow-fed male offspring (n_NC−chow_=11, n_IVF−chow_=12). **B**: Fast glucose of chow and HFD treatment male offspring at 4,10,16 weeks (n_NC−chow_=13, n_IVF−chow_=12, n_NC−HFD_=13, n_NC−HFD_=13). **C**: Fast insulin of chow and HFD treatment male offspring at 4,10,16 weeks (n_NC−chow_=10, n_IVF−chow_=11, n_NC−HFD_=11, n_NC−HFD_=11). **D**: HOMA-IR of chow and HFD treatment male offspring at 4,10,16 weeks (n_NC−chow_=10, n_IVF−chow_=11, n_NC−HFD_=11, n_NC−HFD_=11). **E**: Lipid profile of chow and HFD treatment male offspring at 10,16 weeks (Left:TC, n_NC−chow_=11, n_IVF−chow_=11, n_NC−HFD_=12, n_NC−HFD_=12; Middle:TG, n_NC−chow_=12, n_IVF−chow_=11, n_NC−HFD_=12, n_NC−HFD_=11; Right:LDL, n_NC−chow_=11, n_IVF−chow_=12, n_NC−HFD_=12, n_NC−HFD_=11). *Indicates a significant difference (*P < 0.05*) between NC-Chow and IVF-Chow; #denotes NC-HFD vs. IVF-HFD (*P* < 0.05); ‘ns’ indicates a increased tend in IVF-Chow (0.05 < *P* < 0.1)

IR is one of the high risk factors for NAFLD. To evaluate glucose homeostasis in IVF offspring, PGTTs were performed. The IVF group glucose level had significantly slower recoveries after 30 min, 1 and 2 h of glucose intraperitoneal injection (Fig. 2A) and a larger AUC than the NC group (IVF-Chow vs. NC-Chow: 2316.0 ± 215.0 vs. 1628.0 ± 119.9, *P* = 0.0127). Serum samples were collected at 4, 10 and 16 weeks old to measure fasting glucose (FBG) and insulin (FINS) levels and HOMA-IR (Fig. 2B, C & D). Although FBG levels were similar between the two groups at the ages of 4 weeks, 10 weeks and 16 weeks, the IVF group showed an obvious increasing trend at 16 weeks (*P* = 0.0960). Again, this potential difference was amplified by HFD. The FBG levels in the IVF-HFD group were significantly higher than those in the NC-HFD group at 16 weeks (*P* = 0.0127). FINS levels in the IVF-Chow group were always higher than those in the NC-Chow group (4 weeks: *P* = 0.0327; 10 weeks: *P* = 0.0001; 16 weeks: *P* = 0.0003). Interestingly, the FINS levels in the two HFD-fed groups were similar at 16 weeks but significantly different before (10 weeks, *P* = 0.0001). In addition, HOMA-IR was increased in both the chow and HFD treatments at the ages of 10 weeks and 16 weeks (Chow: 10 weeks, *P* = 0.0056; 16 weeks, *P* < 0.0001. HFD: 10 weeks, *P* < 0.0001; 16 weeks, *P* < 0.0001). These results indicate that IVF offspring developed IGT and IR and even more impaired glucose secretion by HFD.

The liver is not only a central organ for glucose metabolism but also an important site for lipid metabolism. The TG level in the IVF group was considered to be a trend at 16 weeks old (*P* = 0.0945), and the differences were widened further after HFD (10 weeks: *P* = 0.0099; 16 weeks:*P* = 0.0016). There was no difference in TC levels between the two chow-fed groups, and the difference appeared at 16 weeks between the two HFD-fed groups (*P* = 0.0247). The LDL level at 16 weeks, but not 10 weeks, in the IVF-chow group was increased versus the NC group at a trend level (*P* = 0.0996). There was no significant difference until 16 weeks between the IVF-HFD group and the NC-HFD group (*P* = 0.0444) (Fig. 2E).

### Earlier and more severe hepatocellular steatosis and hepatic function in IVF offspring after HFD

**Fig. 3:**
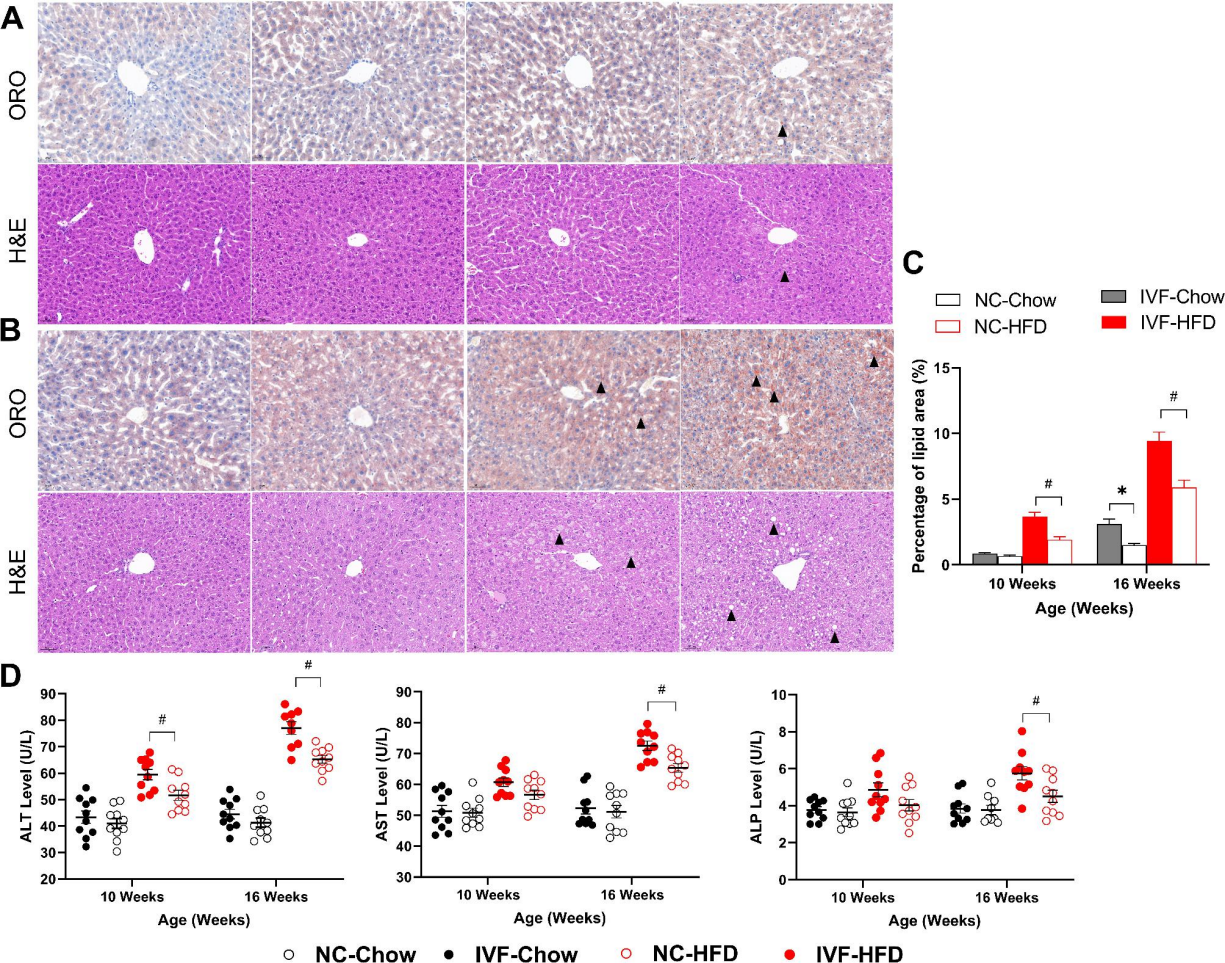
Hepatic lipid accumulation and biochemical indexes. **A**: Liver morphology, ORO and H&E at 10 Weeks (scale bar = 50 μm, from left to right: NC-Chow, IVF-Chow, NC-HFD, IVF-HFD). **B**: Liver morphology, ORO and H&E at 16 Weeks (scale bar = 50 μm, from left to right: NC-Chow, IVF-Chow, NC-HFD, IVF-HFD). **C**: Percentage of lipid droplet area in liver at the age of 10 and 16 weeks. **D**: Hepatic biochemical indexes (Left: ALT, Middle:AST, Right:ALP; n_NC−chow_ =10, n_IVF−chow_ =10, n_NC−HFD_=10, n_NC−HFD_=10). *Indicates a significant difference(*P* < 0.05) between the chow-fed groups, and #indicates a significant difference(*P* < 0.05) between the two HFD-fed groups. ‘ns’ indicates a increased tend in IVF-Chow (0.05 < *P* < 0.1)

Liver samples stained with Oil Red O(ORO) and haematoxylin-eosin(H&E) were used to directly observe lipid accumulation and hepatocellular steatosis, and representative images of those in liver sections are shown in Fig. 3A&B. Although H&E staining revealed hepatic balloon degeneration in both groups after HFD feeding, the lipid size in the IVF-HFD group was increased compared with that in the NC group. Moreover, the relative percentage area of lipid accumulation was also increased in the IVF-HFD group both at the age of 10 weeks (*P* = 0.0281) and 16 weeks (*P <* 0.0001), while it was only increased at 16 weeks in the IVF-chow group (*P <* 0.0001) (Fig. 3C).

To investigate the difference in liver function between the two groups before and after HFD, we measured serum ALT, AST and ALP levels. As Fig. 3D shows, ALT, AST and ALP levels in the IVF group were similar to those in the NC group. The ALT level was significantly increased in the IVF-HFD group both at 10 weeks (*P* = 0.0123) and 16 weeks (*P* = 0.0002) compared with the NC-HFD group. However, compared with the NC-HFD group, the AST and ALP levels in the IVF-HFD group increased significantly only at 16 weeks (AST: *P* = 0.0023; ALP:*P* = 0.0294). These results suggest that IVF offspring were more prone to hepatic steatosis and functional injury after being fed a HFD.

### Upregulated transcription level of AT1R increases hepatic local RAS activity in IVF offspring

**Fig. 4:**
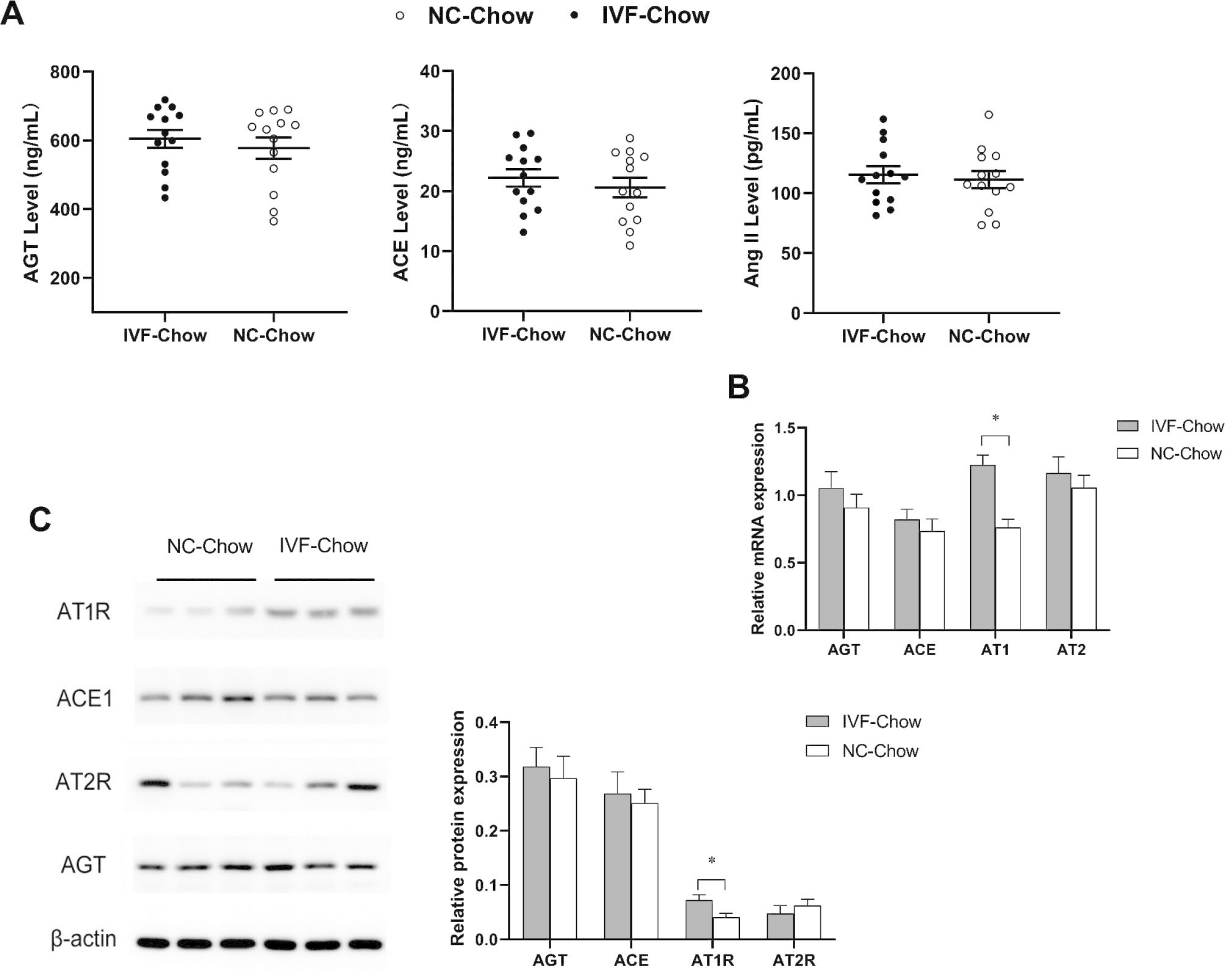
Expression of hepatic local RAS components in male offspring. **A**: serum concentration of circular RAS component in chow-fed male offspring at 4-week-old. **B**: Relative mRNA expression of local RAS component in chow-fed male offspring livers at 4 weeks old. **C**: Representative protein levels of local RAS component in chow-fed male offspring livers at 4 weeks old. *Indicates a significant difference(*P* < 0.05) between the two groups

Systemic and local RASs regulate liver function and liver disease. This role is particularly true for the local hepatic RAS, which remains largely ambiguous in the liver. We measured serum AGT, Ang II, and ACE1 levels, as well as the gene and protein expression levels of AGT, ACE1, AT1R, and AT2R in liver tissue from 4-week-old offspring. As shown in Fig. 4A, serum AGT, Ang II, and ACE1 levels in IVF offspring were similar to those in NC offspring. There were no differences in either the gene or protein expression levels of AGT, ACE1, or AT2R between the two groups. However, the gene (*P < 0.0001*) and protein (*P* = 0.0245) expression levels of AT1R in the IVF group were significantly increased compared with those in the NC group (Fig. 4B&C). These results suggested that abnormal metabolism in IVF offspring livers might be related to upregulated expression of AT1R.

### The AT1R antagonist Losartan has a beneficial effect on the adverse metabolic phenotype after HFD feeding in IVF offspring

**Fig. 5:**
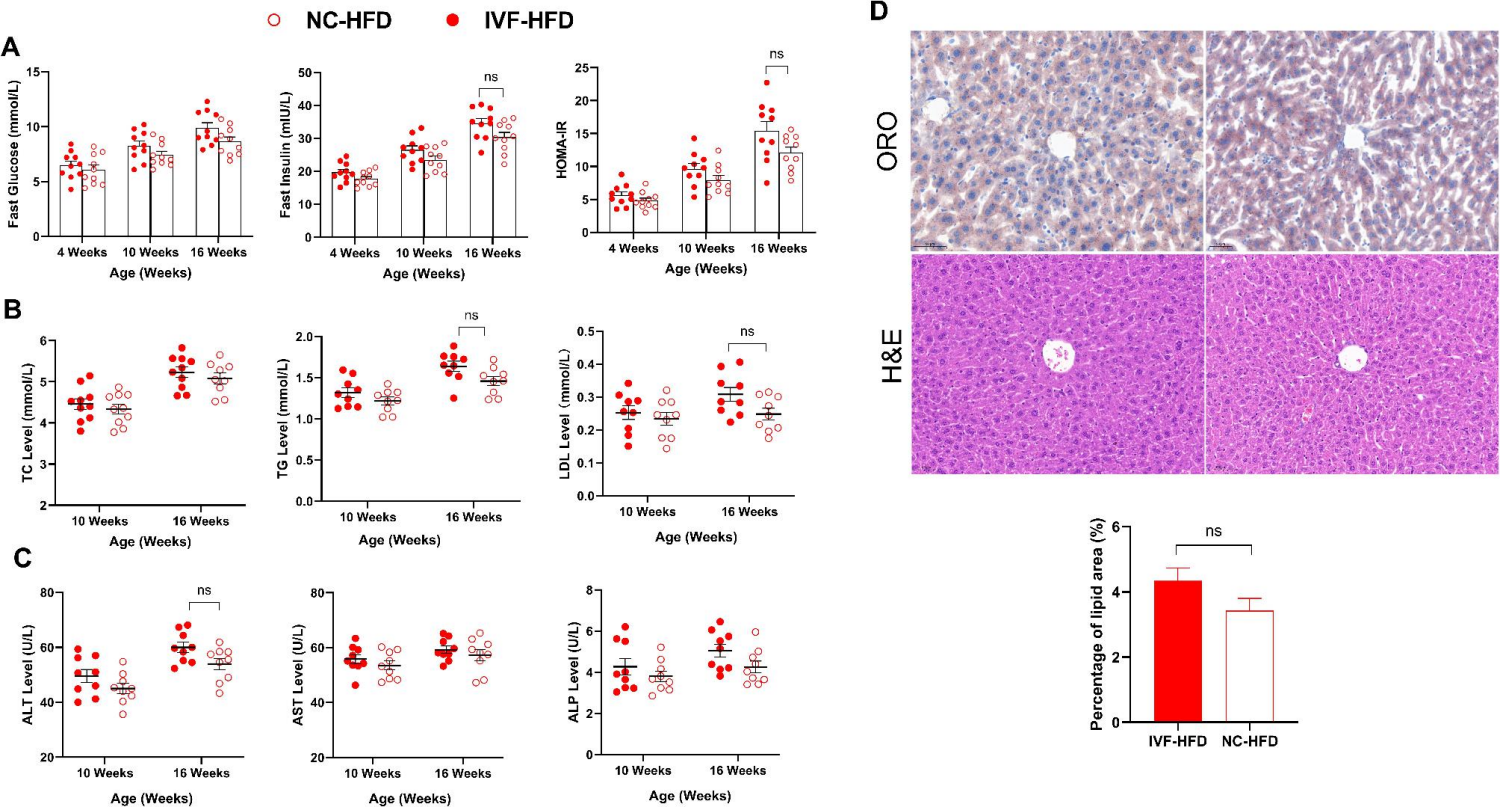
Metabolic phenotype after Losartan administration. **A**: Fast glucose, fast insulin and HOMA-IR in HFD treatment male offspring after Losartan administration at 4, 10, 16 weeks (n = 10 respectively in two groups). **B**: Lipid profile of HFD treatment male offspring at 10,16 weeks after Losartan administration(Left: TC, n = 10 respectively in the two groups ; Middle: TG, n = 9 respectively in the two groups; Right: LDL, n = 9 respectively in the two groups). **C**: hepatic biochemical indexes of HFD treatment male offspring at 10,16 weeks after Losartan administration(Left: ALT, Middle: AST, Right: ALP; all n = 9 respectively in the two groups). ‘ns’ indicates a increased tend levels in IVF-HFD(0.05 < *P* < 0.1). **D**:Representative liver tissue sections were stained with H&E and ORO in HFD treatment male offspring after Losartan administration at 16 weeks (scale bar = 50 μm, from left to right: NC-HFD, IVF-HFD). ‘ns’ indicates a increased tend levels in IVF-HFD(0.05 < *P* < 0.1).

The AT1R antagonist losartan was used to further study the association of higher AT1R expression with abnormal glucose homeostasis and lipid metabolism in IVF offspring. We administered losartan to both HFD groups of offspring from 4 weeks through 16 weeks. Figure 5A shows that the FINS (*P* = 0.0987), as well as the HOMA-IR (*P* = 0.0849), in the IVF-HFD group only had a higher trend level at 16 weeks after losartan administration compared with the NC-HFD group. Moreover, there was almost no difference in FBG between the two groups.

As shown in Fig. 5B, TC levels in the IVF-HFD group were not significantly different from those in the NC-HFD group at either 10 weeks or 16 weeks after losartan intervention. TG (*P* = 0.0563) and LDL (*P* = 0.0839) levels in the IVF-HFD group were not higher than those in the NC-HFD group until 16 weeks, and they were obviously better than those before losartan intervention. Furthermore, after losartan intervention, the significant difference in AST and ALP levels disappeared between the two HFD groups. The increasing trend in ALT (*P* = 0.0874) levels between the two groups were also decreased (Fig. 5C). These results showed that the overexpression of AT1R might also be the main factor contributing to impaired metabolism and hepatic function in IVF offspring.

ORO and H&E staining also showed that the liver lipid droplet size and the relative percentage area of lipid accumulation in the IVF-HFD group decreased after losartan intervention, without a significant difference compared with the NC-HFD group (Fig. 5D).

## Discussion

The present study provides new powerful scientific evidence for the“DOHaD” theory. We used mouse models to address the safety for long-term offspring health of IVF-ET on offspring, which could eliminate the factor of parental infertility. A series of experimental results in this study confirmed for the first time that the IVF offspring had abnormal glucose and lipid metabolism in the liver which was related to the overexpression of local AT1R. Moreover, after HFD consumption, the liver is more prone to lipid droplet accumulation, resulting in hepatocellular steatosis and impaired liver function, leading to a significant increase in the risk of NAFLD.

According to“DoHAD”theory, in addition to adult lifestyle and genetic inheritance, environmental factors in early life, as well as nutrition, can affect the risk of some adult noncommunicable diseases [5, 6]. In the establishment of the model, C57 inbred mice are selected as the embryo donor, which not only ensures genetic stability but is also a preferred choice for studying metabolic diseases. The recipient ICR mice were chosen to ensure a higher pregnancy rate, and the colour of the hair could be used to confirm that the born mice were the offspring of IVF-ET. It is well known that the possible adverse factors of IVF-ET mainly involve the in vitro operation and culture of gametes and embryos, including exposure to hyperoxia, strong light, culture medium composition, and so on. Therefore, we chose a culture environment with a concentration of 5% O_2_, which is close to the oxygen concentration in the embryo environment in vivo, to minimize the oxidative stress damage to the embryo caused by high oxygen exposure. More importantly, more than half of the clinical reproductive centers in the world have chosen to culture human embryos with this oxygen concentration [16]. In addition, our control group selected the offspring of mice that naturally mated and did not receive any intervention during pregnancy to completely mimic the process of human couples giving birth to the next generation during natural pregnancy.

It has been reported in many previous reports that IVF offspring have potential risks of IR and abnormal lipid metabolism in both epidemiological [7, 8] and animal model [9, 10] studies. However, under the appropriate embryo culture conditions, some biochemical indicators of lipid metabolism showed significant differences only after the 16 weeks of age. Therefore, we established a “2-hit” mouse model to understand the differences in glucose homeostasis and liver lipid metabolism between IVF offspring and naturally pregnant offspring. A HFD was used to amplify the potential defects to investigate the risk of disease susceptibility. In addition to being the cause of the amplified potential defects, HFD is also the dietary preference of some people. As reported in the literature, energy and fat intake in children and adolescents increases year by year worldwide [17–19]. According to our research results, among the IVF offspring population, if there is such a dietary preference, the risk of NAFLD is significantly increased. Therefore, it is also worth considering whether the calorie intake control for this population needs to be stricter.

NAFLD is one of the most common chronic liver diseases worldwide and is characterized by increased accumulation of liver fat in individuals who do not drink excessive amounts of alcohol [20]. NAFLD encompasses a spectrum of liver diseases ranging from simple steatosis to nonalcoholic steatohepatitis (NASH), which increases the risk of end-stage liver disease, cirrhosis, and hepatocellular carcinoma [21]. Obesity, IR, type 2 diabetes, abnormal lipid metabolism, vitamin D deficiency, etc., are all high-risk factors that promote the formation of NAFLD [11, 22]. The histological hallmark of NAFLD is the accumulation of triglycerides in hepatocytes, with > 5% of hepatocytes infiltrated with fat [23]. Increased basal insulin levels [6, 24], increased relative liver size and liver lipid accumulation [25] were previously reported in mouse IVF offspring following ET at the two-cell stage in males.Our results show that under normal dietary conditions in the IVF offspring, a slight accumulation of lipid droplets began to appear in the liver at week 16. However, under the stimulation of a continuous HFD, the IVF group showed obvious lipid droplet accumulation at 10 weeks, while the NC group showed similar performance at the age of 16 weeks. The baseline of TG in IVF male offspring has been significantly increased in previous studies, and there were no significant differences in TC changes [6, 24]. In present study, only TG showed an increasing trend at 16 weeks of age with the chow-fed treatment. However, when the defect was amplified by HFD-fed, TG was already significantly elevated at 10 weeks, and there were significant differences in TC and LDL at 16 weeks. In addition, biochemical indicators of liver function, such as ALT, AST and ALP, in the two HFD-fed groups showed significant differences at 16 weeks, indicating that the continuous aggravation of hepatocyte fatty infiltration might damage liver function.

The abnormal growth trajectory of IVF offspring has been reported in many previous studies [26–28]. Our study showed that the body weight of chow-fed IVF offspring began to increase significantly from 8 to 12 weeks, and the liver index began to increase significantly from 14 weeks of age. Patients with NAFLD have been reported to have more pronounced hyperinsulinaemia associated with increased IR in adipose tissue/liver than non-NAFLD populations with similar sex, body weight, and fat [29, 30]. In the present study, fasting insulin levels were consistently significantly higher in the IVF offspring than in the NC offspring, as was the elevated HOMA-IR. In addition, compared with the NC offspring, the levels of TG in the IVF offspring were significantly increased, and the level of LDL showed a rising trend. These results indicate that the IVF offspring themselves do have IR and abnormal lipid metabolism, which generally cause fat accumulation in tissues and organs and may also be the main factor leading to abnormal body weight and liver weight ratios. All of the above results suggest that IVF offspring have high risk factors related to NAFLD, such as body weight gain, hyperinsulinaemia, IR and abnormal lipid metabolism. Exposure to poor dietary habits (HFD) increases the risk of NAFLD.

Many previous studies have reported metabolic abnormalities in IVF offspring, especially in the liver. However, the specific regulatory mechanism is still unclear. In addition to the risk of dyslipidaemia increasing lipid droplet accumulation, the activation of the intrahepatic RAS may also be involved in the progression of NAFLD [31]. Ang II is a key bioactive product of RAS and mainly acts on two types of cell surface receptors, AT1 and AT2. RAS not only regulates cardiovascular and renal homeostasis but is also expressed in a variety of organs, including the liver, which appears to be an important organ involved in the development and progression of NAFLD [32]. Some studies have shown that Ang II promotes the accumulation of TG in the liver, leading to altered plasma lipid utilization [33], decreased fatty acid oxidation in the liver [34], altered LDL secretion [35], and increased new lipogenesis [36]. In addition, activation of RAS stimulates mitochondria to produce more reactive oxygen species (ROS) [37], which is thought to play a key role in the development and progression of NAFLD [38]. In our results, there were no significant differences in serum RAS key components AGT, ACE1 and Ang II between the two chow-fed groups at 4 weeks. There were no differences in the gene and protein expression levels of AGT, ACE1 and AT2R in liver tissues, while AT1R was upregulated in liver tissues of IVF offspring. Previous studies have found that the increased contraction of umbilical cord blood vessels by Ang II in human IVF offspring is due to the increased protein expression and transcription level of AT1R, which is caused by the decreased methylation level of AT1R due to the decreased expression of the methylase DNMT3A [39]. In research on blood pressure regulation and vascular function of IVF offspring, unbalanced expression of AT1R and AT2R receptors was found in mice, resulting in an enhanced vascular contraction response to AngII [40]. Our study is the first to confirm that the upregulation of local AT1R in IVF offspring livers enhances the activity of local RAS in the liver.

AT1R blockers could prevent the progress of liver injury induced by D- galactosamine in rats with liver failure, resulting in the improvement of survival rate, the decrease of liver enzymes, the decrease of liver histopathology and tissue-specific inhibitors of metalloproteinases [41]. AT1R antagonists have also been shown to decrease AST levels and inhibit hepatic stellate cell activation, oxidative stress, transforming growth factor beta 1 expression, and hepatic fibrosis [42]. In the present study, losartan, an AT1R blocker, was able to significantly reduce the AST level in the offspring of the two groups after HFD and improve hepatocyte steatosis and lipid metabolism. In addition, another important finding in this study was that AT1R blockade could almost eliminate or significantly reduce the differences between the IVF group and NC group in most of the biochemical indicators related to IR, liver fat metabolism and liver function after HFD treatment. In other words, the abnormal metabolism of glucose and lipids in the liver in the IVF group was mostly caused by the differential expression of AT1R.

## Conclusion

In summary, the evidence presented in the present study indicates that the expression levels of the AT1R gene and protein in IVF-conceived mouse livers were changed. Modifications to DNA methylation may be involved in the regulation and thus the long-term health of IVF-conceived offspring. Given the widespread use of ART, it is important to further explore the genetic and regulatory mechanisms underlying its long-term effects on the health of offspring. These findings offer important new insights into the original embryogenic mechanisms of metabolic disease in adults. This allows us to seek novel ways to minimize ART-induced metabolic dysfunction from childhood through adulthood in later generations.

## Electronic supplementary material

Below is the link to the electronic supplementary material.

**Additional file 1**

## Data Availability

The data that support the findings of this study are available from the corresponding author upon reasonable request.
