# Supplementary material for: Altered local RAS in the liver increased the risk of NAFLD in male mouse offspring produced by in vitro fertilization
Source: BMC Pregnancy Childbirth. 2023 May 12;23:345. doi: 10.1186/s12884-023-05681-8 (PMC10176674; doi:10.1186/s12884-023-05681-8)

Supplementary figure 1. Western Blot gels of AT1R

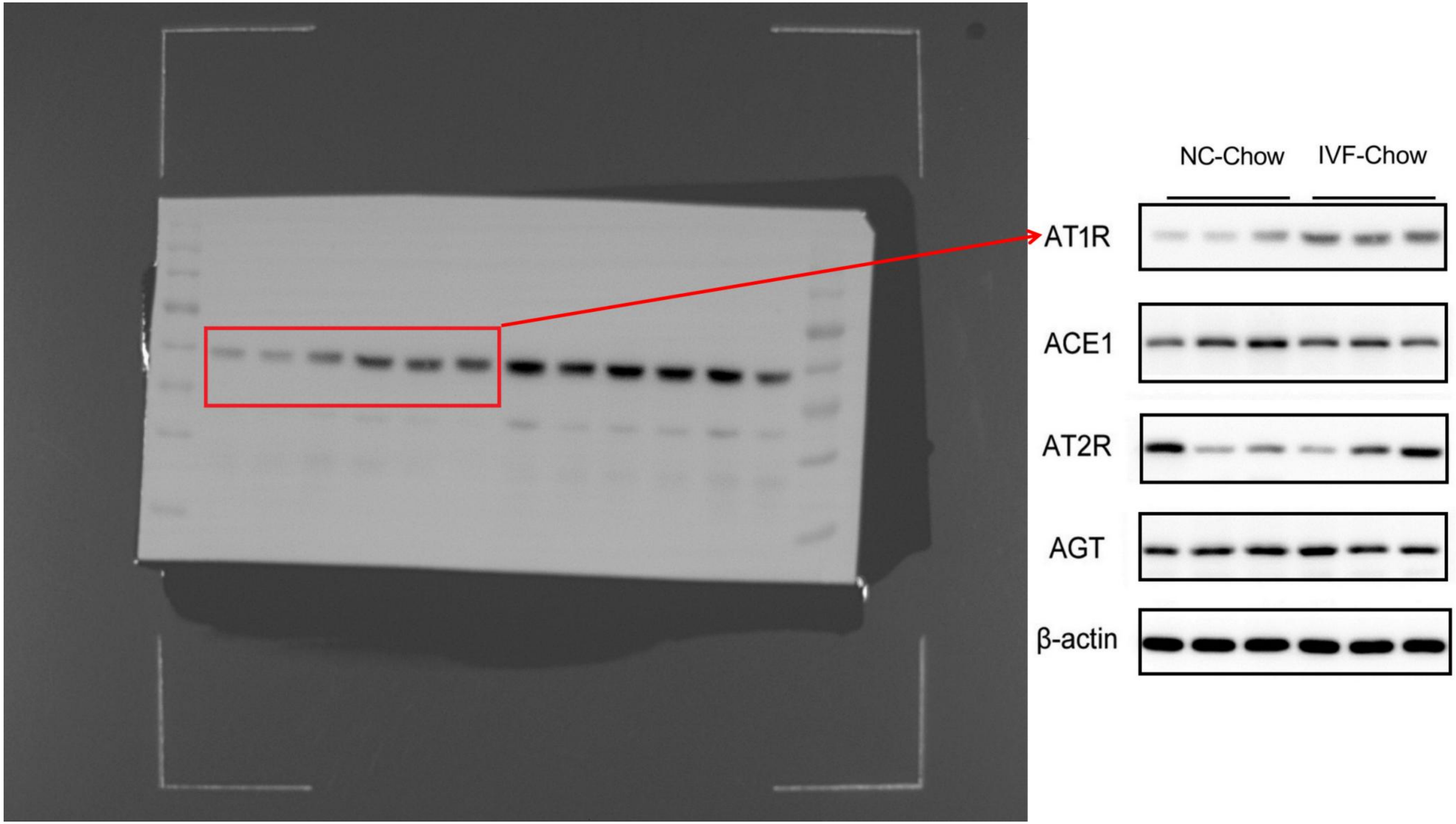

Supplementary figure 2. Western Blot gels of ACE1

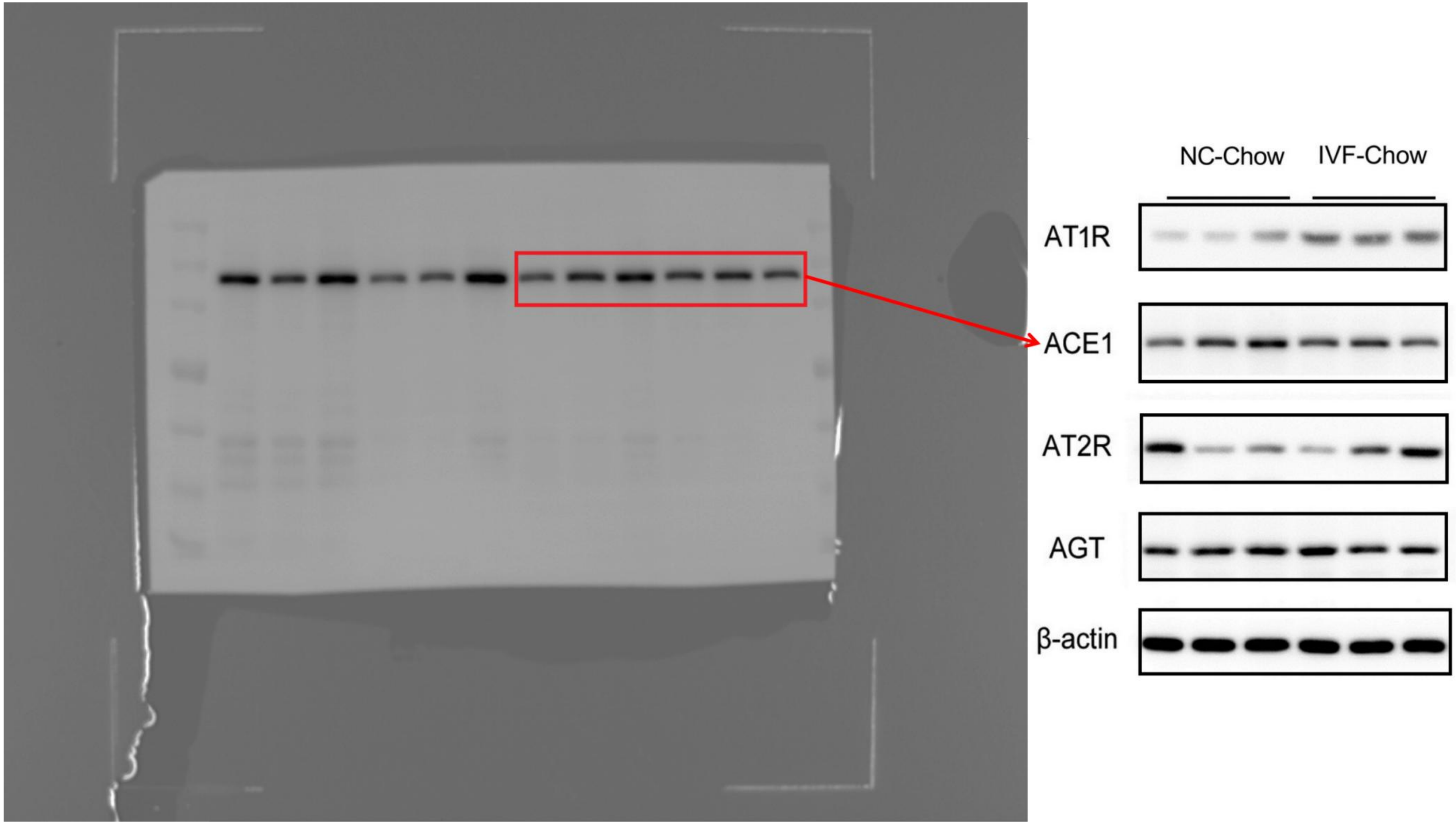

Supplementary figure 3. Western Blot gels of AT2R

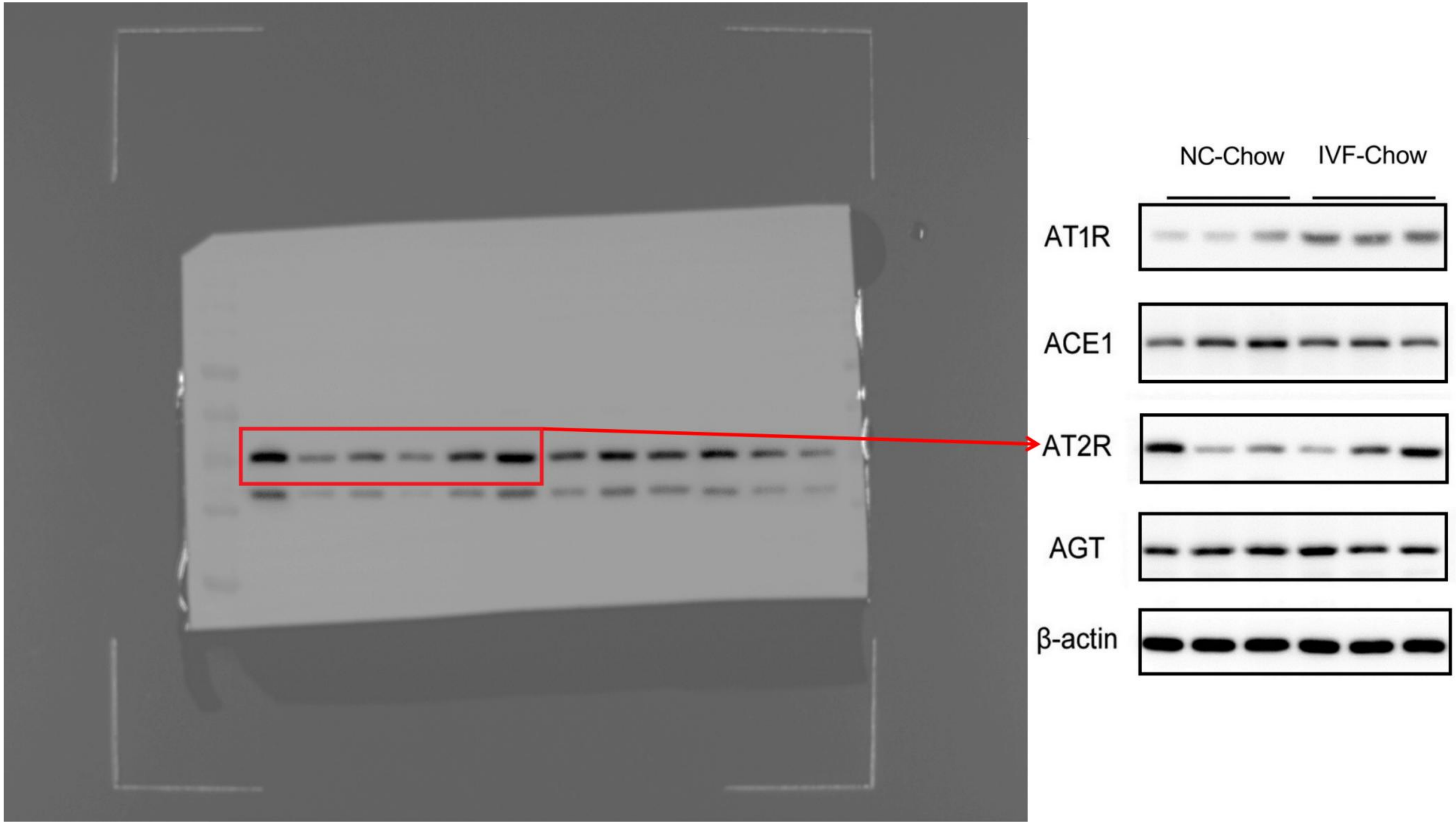

Supplementary figure 4. Western Blot gels of AGT

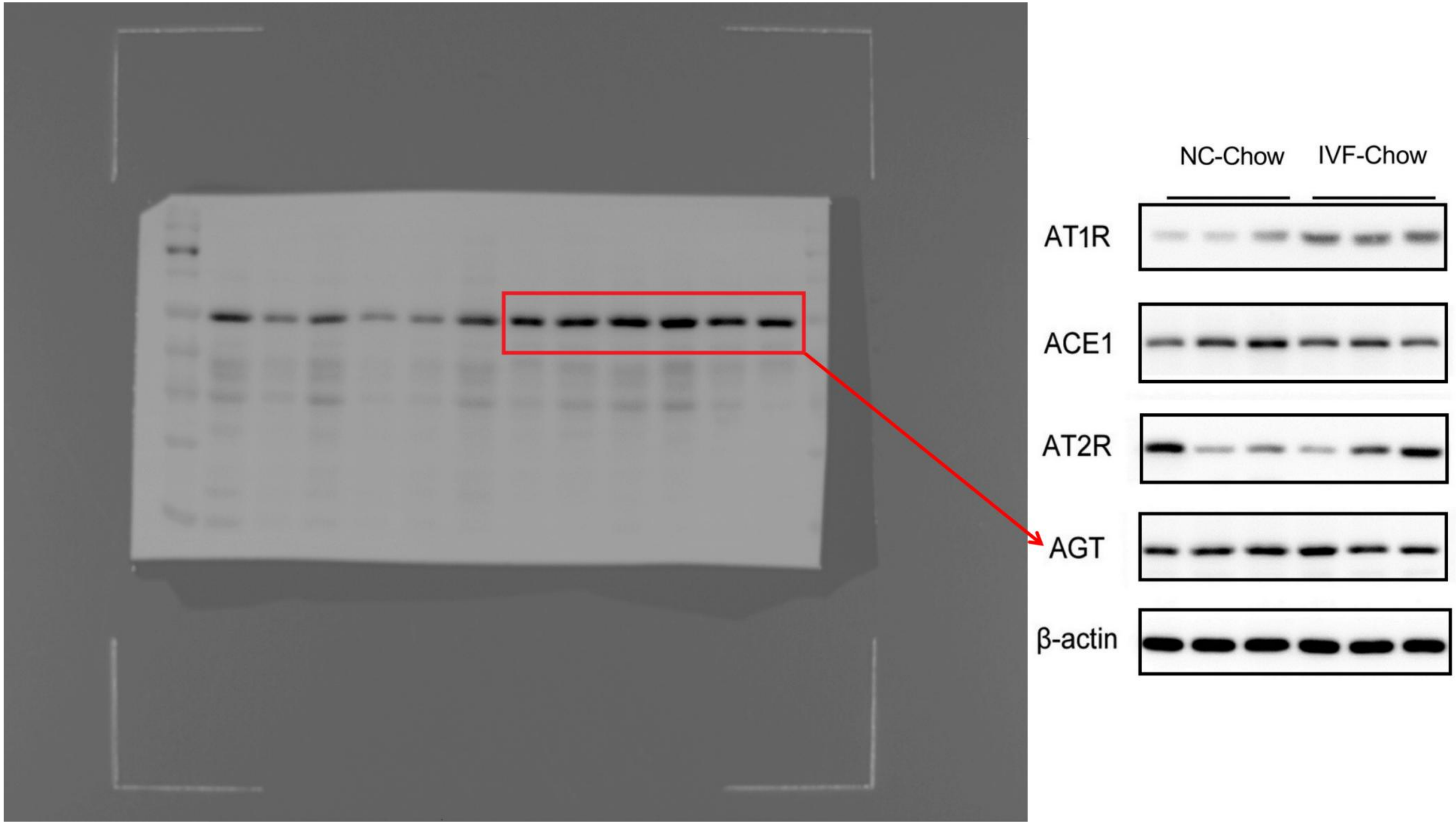

Supplementary figure 5. Western Blot gels of β-actin

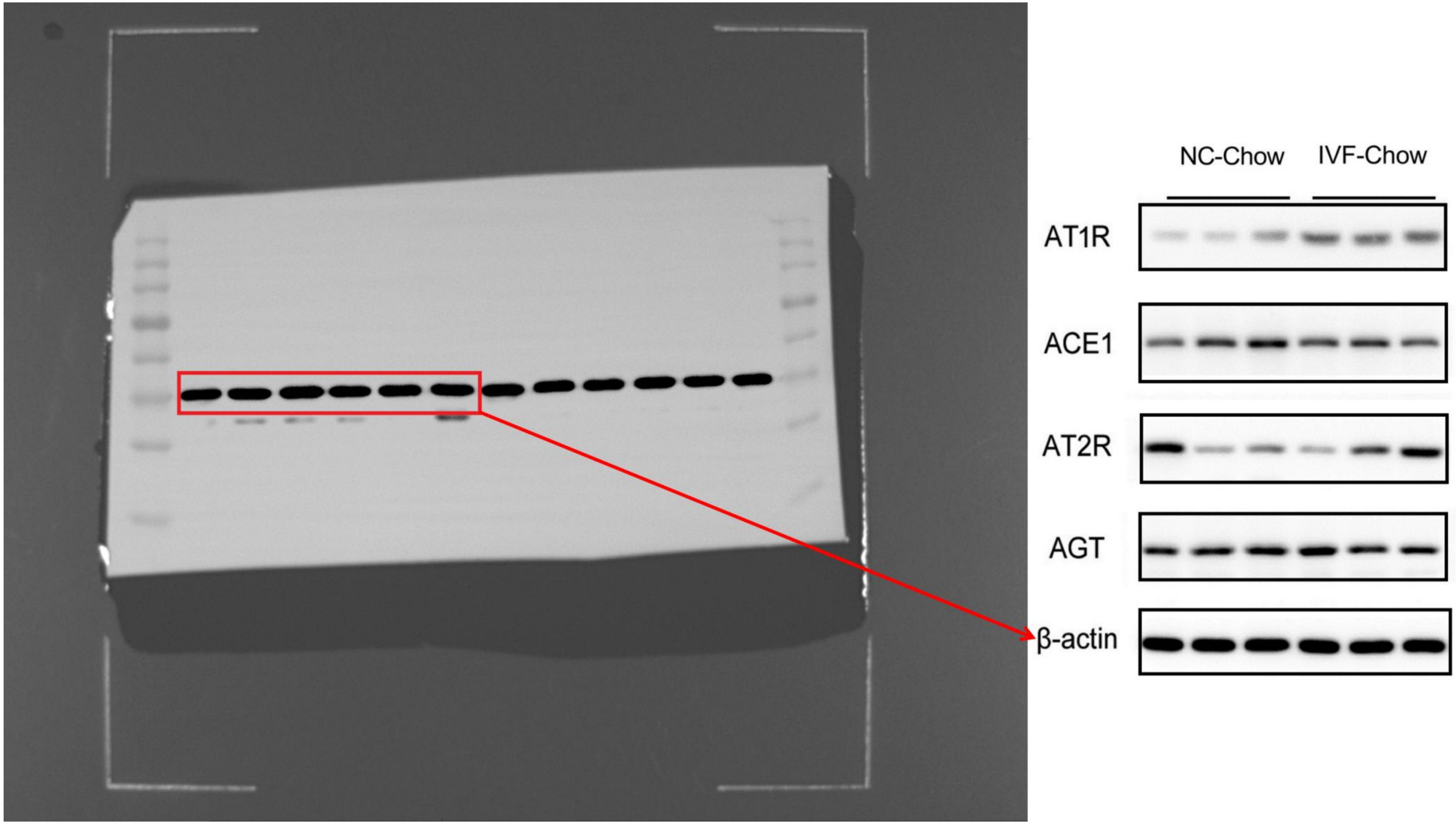

Supplement: Supplementary file 1 — Additional file 1 [file 12884_2023_5681_MOESM1_ESM.pdf]
